# What We Owe to Experiments on Animals

**Published:** 1904-01-16

**Authors:** Stephen Paget


### Hospital Clinics and Medical Progress.

### WHAT WE OWE TO EXPERIMENTS ON ANIMALS.

By Stephen Paget.

### *II.—PATHOLOGY, BACTERIOLOGY, AND THERAPEUTICS.*

(*Continued from page* 255.)

#### VI.—Rabies.

Pasteur, in his first experiments with rabies (hydrophobia), used the saliva of rabid animals for his inoculations. Here is a good story of that period of his work :—

"The rabid beast was a huge bulldog, foaming at the mouth and howling in his cage. All attempts to induce the animal to bite, and so infect one of the rabbits, failed. 'But we *must*,' said Pasteur, 'inoculate the rabbits with the saliva.' Accordingly, a noose was made and thrown, the dog secured and dragged to the edge of the cage, and his jaws tied together. Choking with rage, the eyes bloodshot, and the body convulsed by a violent spasm, the animal was stretched on a table and kept motionless while Pasteur, leaning over his foaming head, sucked up into a narrow glass tube some drops of the saliva."

Later, instead of saliva, he used an emulsion of the brain or of the spinal cord, because the disease has a special action on these structures. These inoculations gave better results, but they were of very little help toward the discovery of a preventive treatment, because they did not fix or control the varying incubation period of the disease. Therefore he changed the method of inoculation, and put the virus not under the skin, but in its proper environment, in the tissues which are its natural home, which it picks out for itself. These inoculations under the *dura mater* were the turning point of all his work. Roux tells a story of the first of them :—

"Next day, when I informed Pasteur that the intra-cranial inoculation had offered no difficulty, he was moved with pity for the dog. 'Poor beast, his brain is doubtless injured; he must be paralysed.' Without reply I went down to the basement to fetch it, and let it come into the laboratory. Pasteur did not like dogs, but when he saw this one, full of life, inquisitively rummaging about in all directions, he exhibited the greatest delight and lavished most charming words upon it."

By sub-dural inoculations, transmitting the virus through a series of rabbits, he obtained the *virus fixe*, having a definite and unalterable incubation period of six days, neither more nor less. He could increase the power of the virus by continued transmission through rabbits; he could decrease its power, by drying the animals' cords after death. A cord thus kept and dried loses virulence slowly and steadily, day by day; at the end of a fortnight it is wholly non-virulent. Thus he could always have at hand a complete graduated series of cords, one for each day of the fortnight, ranging from absolute non-virulence up to the full virulence of the *virus fixe*.

The preventive treatment of rabies, from the first case (Joseph Meister, July, 1885) to the present day, is founded on this exactly graduated series of virulences. By way of illustration, we have the familiar fact that a patient after scarlet fever is protected against scarlet fever: the "toxin" of the fever has brought about a natural "antitoxin" in the patient, safeguarding him against a second infection. So it is with the Pasteur treatment against rabies. A minute quantity of cord-substance, rubbed up in a little fluid, is injected under the skin. The treatment begins with non-virulence, and proceeds day by day, steadily and equably, through all the exact and minute gradations of virulence. Every day, and all day long, the patient is brewing in his own body a natural "antitoxin" against the "toxin" of the treatment, and as the strength of the toxin is increased so is the strength of the antitoxin. But the full strength of this natural resistance is not attained till 14 or 15 days have elapsed after the end of the treatment. This fact has been proved by experiment on animals. A fortnight after his last and strongest dose of toxin the patient is full-up, if the phrase may be forgiven, with antitoxin. The original source of infection, the bite itself, which was lying dormant all this time, has been outwitted and outstripped; it has lost its one chance, it has overslept itself; it could do nothing, even if it tried to wake now, in a body saturated with antitoxin. The patient has been hard at work while it slept; he is self-protected, self-immunised, self-antitoxinised.

With slight modifications, according to the gravity of the case, such is the treatment at the Pasteur Institute. A fuller account of it, with references, reports, etc., is given in the book, "Experiments on Animals." In the returns published by the Institute, those cases are excluded which died of rabies during treatment or within fifteen days of the end of the treatment. They are counted as having come too late: the original infection awoke before the patient was fully immunised. They are not numerous: *e.g.*, 2 in 1897, 1 in 1898, 6 in 1899, 7 in 1900, 3 in 1901, and 1 in 1902. The exclusion of such cases is fully justified by the evidence of bacteriology; and they are excluded from the tables that are quoted here.

Before Pasteur, what was the mortality among people bitten by really mad dogs? How many of them died of hydrophobia? Most of them escaped —*The man recovered from the bite, The dog it was that died.* Leblanc guessed it to be 16 per cent.: Pasteur guessed between 15 and 20 per cent. Let us, for the sake of argument, guess it at 10 per cent. What is it now? Here are the figures from the Pasteur Institute :—

| Year. | Patients treated. | Deaths. | Mortality p c. |
|---|---|---|---|
| 1886 | 2,671 | 25 | 0 94 |
| 1887 | 1,770 | 14 | 0·79 |
| 1888 | 1,622 | 9 | 0 55 |
| 1889 | 1,830 | 7 | 0 38 |
| 1890 | 1,540 | 5 | 0 32 |
| 1891 | 1,559 | 4 | 0 25 |
| 1892 | 1,790 | 4 | 0 22 |
| 1893 | 1,648 | 6 | 0·36 |
| 1894 | 1,387 | 7 | 0 50 |

| Year. | Patients treated. | Deaths. | Mortality p.c. |
|---|---|---|---|
| 1895 | ... 1,520 | ... 5 | ... 0·33 |
| 1896 | ... 1,308 | ... 4 | ... 0·30 |
| 1897 | ... 1,521 | ... 6 | ... 0·39 |
| 1898 | ... 1,465 | ... 3 | ... 0·20 |
| 1899 | ... 1,614 | ... 4 | ... 0·25 |
| 1900 | ... 1,420 | ... 4 | ... 0·28 |
| 1901 | ... 1,321 | ... 5 | ... 0·38 |
| 1902 | ... 1,105* | ... 2 | ... 0·18 |

* This falling-off in the number of cases at Paris is explained by the establishment of similar institutes at Lille, Marseilles, Montpellier, Lyon, and Bordeaux.

That is to say, the mortality, instead of 10 per cent., is less than 1 per cent.

But these figures raise two questions :—

1. Who can tell how many of these patients went home, and were lost sight of, and died of hydrophobia at home ?

2. How many of them were bitten by animals that were really rabid ?

1. It must be admitted that some deaths might occur and might not be reported to the Institute. It is the custom of the Institute to ask the medical men in charge of cases after treatment to report any failure. And it is not likely that many cases in civilised districts would go unreported, seeing that an Institute patient must be an object of general interest to his neighbours, and seeing that a case of hydrophobia is not easily erased from the memory. A few months ago (May 1903), the Institute, in its annual report on the treatment, was careful to note and to emphasise the case of a carpenter in a Welsh village who had died of rabies nearly two years after treatment. This does not look as though it neglected to keep in touch, so far as possible, with its old patients.

2. All patients treated at the Institute are divided into three classes, as follows :—A. The rabies of the biting animal was experimentally proved by the development of the disease in animals bitten by it or inoculated with its cord. B. The rabies of the biting animal was proved by veterinary examination. C. The biting animal was suspected of rabies. We shall all agree that the A patients were bitten by animals that were really mad. The grand total of A patients, from 1886 to 1902, is 3,667, of whom 28 died. That is to say, the deaths were not 10 or 15 per cent., but less than 1 per cent. Here are the figures for 1902 :—

| | Bites on the Head. | | | Bites on the Hands. | | | Bites on the Limbs. | | | Total. | | |
|---|---|---|---|---|---|---|---|---|---|---|---|---|
| | Treated. | Deaths. | Mortality | Treated. | Deaths. | Mortality. | Treated. | Deaths. | Mortality. | Treated | Deaths | Mortality. |
| | | | Per cent. | | | Per cent. | | | Per cent. | | | Per cent. |
| Class A .. .. .. .. .. .. | 20 | — | — | 87 | 2 | 2·30 | 43 | — | — | 150 | 2 | 1·33 |
| Class B .. .. .. .. .. .. | 51 | — | — | 405 | — | — | 169 | — | — | 625 | — | — |
| Class C .. .. .. .. .. .. | 21 | — | — | 190 | — | — | 119 | — | — | 330 | — | — |
| Total .. .. .. .. .. | 92 | — | — | 682 | 2 | 0·29 | 331 | — | — | 1,105 | 2 | 0·18 |

There is no room here for results from other Institutes, or for any full statement of the whole subject. All these things will be found in the Annals of the Institute published every month, or in the reports of other Institutes, etc. Let us see what is said against the treatment, by the opponents of all experiments on animals, and what they recommend in its place.

1.

An anti-vivisection society has lately published a paper entitled, *The Pasteur "Cure" for Rabies: 1,857 reported deaths! Revised to January 1st*, 1903. Let us examine this paper. It covers a period of 17 years. It ranges over the whole world —Japan, Cochin-China, Circassia, Russia and Caucasia, Algeria, Spain and Portugal, Bessarabia, North and South America, and everywhere else. It charges its deaths on the diverse Pasteur Institutes of France, Italy, Germany, Austria, Russia, and India, and on Athens, Cairo, Budapest, Constantinople, Samara, Batavia, Kasauli, Tananarivo, Tokio, Buenos Ayres, Chicago, New York, etc. In a word, it spreads its deaths very thin—over 17 years of time and all inhabited space. By this method, one might easily collect the same number of deaths after any operation of surgery, and publish them in a paper entitled, *Victims of the Craze for Operating*.

But let us look into these deaths more closely. The paper is elaborate, but it is not very critical :—

1. It quotes among its "sources of information," *L'Ami du Peuple*, *L'Intransigeant*, *Le Petit Journal*, *La Croix*, *Le Patriote*, *Le Petit Parisien*, the *China Gazette* (quoted in the *New England Anti-vivisection Society Monthly*), and a thing called *The Dog Question*, published at Dobbs Ferry, New York. Really, what *Le Petit Parisien* says is not evidence in this difficult statistical inquiry.

2. It quotes, with amazing frequency, one little French journal, which is hardly recognised by the medical profession over here. The authority of this one insignificant journal is invoked 55 times over the first 200 cases. Of course, there is an anti-Pasteur journal, with an anti-Pasteur editor, just as there is an anti-antitoxin journal. Let us suppose that a Society exists for the Abolition of the Police Force, and that it publishes a list of charges of inefficiency against that excellent body of men. If 55 out of the first 200 charges were made on the authority of a single Sunday-school magazine, should we not be rather surprised ? Moreover, the same anti-vivisection society that publishes *The Pasteur Cure for Rabies* also publishes an "official journal" called the *Zoophilist*, which simply worships this little French journal, and says : "*We gladly endorse its vehement protest against the filthy nostrums for inoculation which emanate from the Pasteurian factory of Paris*" (*Zoophilist*, December 1903).

3. It quotes cases without names, without ages, without saying what sort of animal bit them, when they were bitten, when they were treated, or when they died. There is nothing but a bare reference. For example, here are two entries, side by side in this paper. *Four persons. Treated at Turin.* 1890. *Relazione del Servizio Batteriologico, Turin,* 1896. *Eight persons, Hungary,* 1890. *Statistik des Budapester Institutes for* 1897. It piles up scores of cases in this way, leaving us to guess how many of them would be admitted as evidence by a committee of experts.

4. Worst offence of all, it does not say how long the treatment lasted. It does not face the question, whether the patient died within 15 days of the end of the treatment. It gives the date of the *first* inoculation, and the date of the death ; but it does *not* give the date of the *last* inoculation. Thus, it sweeps into its net all the cases where death occurred before the immunity was complete.

Let us, for the sake of argument, assume that 20 per cent. of the deaths recorded in this paper were indeed due to failure of the treatment. That gives us, in 17 years, 371 deaths for the whole world; say, 22 deaths *per annum.* Of these 22 deaths, shall we assign one death, or decimal point something, to the charge of the one great Institute at Paris ?

Those who doubt whether the treatment can indeed immunise men, women, and children, do not deny that it can immunise animals. And, if they still refuse to believe in human immunity, they must explain away the fact that every patient receives, at the close of the treatment, doses of toxin that would otherwise bring about an immediate general massacre of the innocents in every Institute all over the world, from France to Japan, and from St. Petersburg to Algiers.

2.

The *Buisson Bath for the Prevention and Cure of Hydrophobia* is recommended, by anti-vivisection societies, as the real and proper treatment. "The Buisson treatment," says the *National Canine Defence League,* "is founded upon the simple common-sense principle that if poison is injected into a person's veins the best thing to do is to get it out as quickly as possible." And, for further particulars, it refers us to a retired naval officer in Surrey. This gift of healing can be obtained either at Norwood, or at the "National Anti-vivisection Hospital," or in India. Let us put aside our natural prejudice against experiments on hospital patients, and try to take the Buisson Bath seriously.

Dr. Buisson, who invented it, believed that he had hydrophobia, and took a vapour-bath to kill himself. *And at* 42° (127 *F.*) *I was cured.* It was, of course, an ordinary case of imagination of a disease. The "simple common-sense principle" of the bath has been tested on inoculated rabbits, without any result : and the statements quoted from India are mere rubbish. "A mass of cures in Asia" is mentioned ; but only one case is quoted, and nothing is said about the dog that bit that solitary patient. Also the case of Pauline Kiehl is quoted, who was "refused treatment by M. Pasteur." In India, they use the bath anyhow ; for snake-bite, for cholera, for a case that may be "*interpreted as one of tetanus rather than hydrophobia.*" Happily it has killed nobody, not even M. Buisson ; and a warm bath is doubtless acceptable to Indian natives in sickness or in health. But it would be a shame, if this idiotic bath treatment were ever used on some ignorant person who was in real danger of hydrophobia. At the best, it would be a wicked experiment on a human being : at the worst, it would be something very like manslaughter.

(*To be continued.*)

